# 

**Published:** 1813-09

**Authors:** 


### MONTHLY CATALOGUE OF MEDICAL BOOKS.

OBSERVATIONS on Indigestion, in which is satisfactorily shown the Efficacy of Ipecacuan, relieving this, as well as its connected train of Complaints, peculiar to the Decline of Life. By Mr. Daubenton. The fourth edition, with additional notes and observations. By Alex. P. Buchan, M.D. 12mo. boards.—Callow.

Appendix to Observations on the Contracted Intestinum Rectum; containing some additional Facts relative to that Complaint, with several Cases, and two Engravings. By W. White. 12mo.—Callow.

A Familiar Treatise on Cutaneous Diseases, exhibiting a popular View of their respective Symptoms, detailing the Limits of secure Self-treatment, and illustrating the perilous Abuse of Indiscriminate Remedies. By John Wilson. 8vo.—Wilkie and Robinson.

### *NOTICES TO CORRESPONDENTS.*

*Dr. Spark's letter arrived too late for insertion this month.—A very important paper upon Medical Reform, signed "a disinterested Physician," was only deferred that it might appear entire: it will commence the next Number, and we would especially invite our readers' attention to it.—Mr. Hemingway requests Obstetricus to peruse the 355th page of Burn's Principles of Midwifery, when he will find the diagnostic symptoms of puerperal convulsions to accord very much with Mr. H.'s ideas.—A professional Correspondent wishes to be informed whether he is to understand that the seventeen gallons of fluid which were extracted by Mr. Chevalier in his singular case of Ovarial Dropsy, related in the last Number of this Journal, were wine or beer gallons?*

